# Adipokines, C-reactive protein and Amyotrophic Lateral Sclerosis – results from a population- based ALS registry in Germany

**DOI:** 10.1038/s41598-017-04706-5

**Published:** 2017-06-29

**Authors:** Gabriele Nagel, Raphael S. Peter, Angela Rosenbohm, Wolfgang Koenig, Luc Dupuis, Dietrich Rothenbacher, Albert C. Ludolph

**Affiliations:** 1 Institute of Epidemiology and Medical Biometry, Ulm University, Ulm, Germany; 2 Department of Neurology, Ulm University, Ulm, Germany; 3 Department of Internal Medicine II - Cardiology, University of Ulm Medical Centre, Ulm, Germany; 4 Deutsches Herzzentrum München, Technische Universität München, Munich, Germany; 5 DZHK (German Centre for Cardiovascular Research), Partner site Munich Heart Alliance, Munich, Germany; 6 INSERM U1118, Université de Strasbourg, Strasbourg, France

## Abstract

To investigate the associations of leptin, adiponectin and high-sensitive (hs) C-reactive protein (CRP) with risk and prognosis of amyotrophic lateral sclerosis (ALS). Data from a population-based case-control study in Southern Germany (10/2010–6/2014) of 289 ALS patients (mean age of 65.7 (SD 10.5) years, 59.5% men) and 506 controls were included. During median follow-up of 14.5 months of 279 ALS patients 104 (53.9% men, 68.9 (10.3) years) died. Serum samples were measured for leptin, adiponectin and hs-CRP. Conditional logistic regression was used to estimate ALS risk. Survival models were used to appraise the prognostic value. ALS patients were characterized by lower levels of school education, BMI and smoking prevalence. Adjusted for covariates, leptin was inversely associated with ALS risk (top vs. bottom quartile: OR 0.49; 95% CI 0.29–0.80), while for adiponectin a positive association was found (OR 2.89; 95% CI 1.78–4.68). Among ALS patients increasing leptin concentrations were associated with longer survival (p for trend 0.002), while for adiponectin no association was found (p for trend 0.55). For hs-CRP no association was found. Leptin and adiponectin, two key hormones regulating energy metabolism, were strongly and independently related with ALS risk. Leptin levels were further negatively related with overall survival of ALS patients.

## Introduction

Amyotrophic lateral sclerosis (ALS), the major adult-onset motor neuron disease, is characterized by the progressive loss of motor neurons and leads to death within 3–4 years after diagnosis, usually from respiratory insufficiency^1, 2^. Incidence of ALS is between 2 and 4 per 100.000 person years^1^ and we recently reported an incidence of 2.5 per 100.000 person years in the South-West of Germany within a study with a capture-recapture rate of 82%^3^. Multiple genetic risk factors and environmental and lifestyle factors are postulated to contribute to disease pathogenesis^1^.

A large number of clinical evidence implicates dysfunctional energy homeostasis in ALS^4^. First, decreased weight appeared as a strong negative prognostic factor in retrospective studies^5–7^. Second, various biomarkers of metabolism^8^, including circulating lipids^9, 10^, adipose tissue distribution^11^ or cardiovascular health^12, 13^ were associated with survival of ALS patients or disease prognosis. A study on metabolomics revealed different metabolic profiles in ALS patients and controls^14^. Third, one retrospective study found a negative association between type 2 diabetes and survival in ALS patients^15^ but this finding was not consistent with other studies^16^. Existing clinical evidence is further reinforced by recent epidemiological studies showing that ALS onset and mortality are associated with low body mass index (BMI)^17, 18^ and lower co-occurrence of type 2 diabetes in European countries^19, 20^. Weight loss and energy imbalance appears as a direct contributor to neurodegeneration in ALS animal models, since high fat feeding was able to improve survival and delay neurodegeneration of ALS in mice^21^. Furthermore, a recent clinical trial suggested that a hypercaloric diet prolonged lifespan of ALS patients under gastrostomy^10, 22^. Altogether, these studies suggest that an alteration of energy metabolism might be an important driving force in ALS.

Relevant biomarkers of an individual’s energy status are circulating levels of several adipokines as hormones secreted by the adipose tissue in direct correlation with their energy stores. For instance, leptin secretion is generally positively correlated with size of the lipid droplet of the adipocyte^23^, while adiponectin levels are decreased with adiposity^23^. Both of these adipokines signal in the hypothalamus to trigger antagonistic effects, *ie* satiety and increased energy expenditure for leptin, and weight loss and increased energy intake for adiponectin^23^. Besides adipokines, systemic low grade inflammation, indirectly measured by high-sensitive (hs) C-reactive protein (CRP) levels, may also be associated with dysfunctional metabolic homeostasis, and could modulate adipokine secretion and action.

The objective of the present study was to analyze the associations of leptin, adiponectin and hs-CRP as biomarkers of energy metabolism and systemic low-level inflammation with the risk of ALS in a population-based case-control study conducted in southern Germany after controlling for potential confounders. In addition, in patients with ALS the prognostic value of the respective blood markers for overall survival was investigated in a cohort approach.

## Material and Methods

### Study design and study population

The ALS registry Swabia has been described previously in detail^3, 24, 25^. In brief, it is a clinical-epidemiological registry in a defined geographic region in the South-West of Germany, to estimate incidence and to describe the natural history of ALS. The catchment area consists of the region of Swabia with approximately 8.4 million inhabitants.

From October, 2010 until June 2014 all newly diagnosed ALS cases were registered prospectively^25^. Patients were also asked to provide informed consent to participate in a population-based case-control study in order to investigate risk factors of ALS. ALS cases were defined by the diagnosis of possible, probable or definite ALS according to the revised El Escorial criteria^26^. The following sites of onset were distinguished: bulbar, cervical, thoracic, and lumbosacral. Signs of upper or lower motor neurons damage were also recorded. All cases were reviewed by an experienced neurologist according to pre-defined standardized criteria^26^. Notifications of patients with suspected ALS were tracked and evaluated by the registry personnel during the clinical course of disease.

For each case (total N = 289), we intended to match two sex and age matched control subjects randomly sampled from the general population as registered in the regional registry office (“Einwohnermeldeamt”) of the catchment area of the case as specified by the postal code. The control subjects were contacted by mail. After informed consent study nurses visited cases and controls for an identical, standardized interview, neuropsychological testing, and blood sampling. Response rates were 65% in cases (20% refused and 15% could not be contacted) and 19% in controls (39% refused and 42% did not respond after several attempts to get in contact per mail and telephone). Most frequent reasons for refusal were ‘lack of interest’, followed by’limitations due to ill health or age’ and ‘lack of time’.

Non-fasting blood samples were collected and processed according to a standardized protocol and transported in cooled containers to the study center. Serum was obtained by centrifugation for 10 min at 2000 RPM × g and 4 °C (Heraeus Multifuge 3 S-R, Fa. Thermofischer). Blood specimens were transferred into 0.5–1.0 ml sample containers with screw tops on the same day and were stored at in −80° Celsius freezers until further analysis. For the current study all consecutively registered cases until June 2014 (N = 289) and matched controls (N = 506) with blood sample were selected.

In addition, ALS patients were actively followed-up and annually interviewed. Furthermore, record linkage with the central registration office in Baden-Württemberg and the local registration offices in Bavaria were performed. In case of death the date was obtained from the local registration offices. Survival times were censored at the date of the last systematic mortality update (April 30, 2014). Since 10 ALS cases were recruited after the last mortality update, their data was excluded for the analyses of the prognostic value of the biomarkers.

### Ethics Statement

International, national and state rules were followed implementing the ALS registry Swabia. We obtained full ethical approval of the ethical committees of Ulm University and the regional medical associations (“Landesärztekammer Baden-Württemberg” and “Landesärztekammer Bayern”).

### Biomarker measurement

Serum leptin (ng/ml) was measured by enzyme linked immunosorbent assay (ELISA) (Qantikine^®^, Human Leptin Immunoassay, R&D Systems, Wiesbaden, Germany). The lower detection limit (LOD) of leptin was 0.078 ng/ml. The interassay coefficient of variation (CV) was 5.94%. Serum levels of adiponectin (µg/ml) were also determined by a commercial ELISA (Qantikine^®^ Elisa, Human Total Adiponectin/Acrp 30 Immunoassay, R&D Systems, Wiesbaden, Germany). The LOD was 0.246 ng/ml and the interassay CV was 2.55%. Hs-CRP in serum was measured by a latex-enhanced high-sensitivity immunonephelometry assay on a Behring Nephelometer II (CardioPhase^®^, Siemens). The LOD was 0.17 mg/L and the CV was 3.31%. All laboratory analyses were performed in blinded fashion at the research laboratory of the Department of Internal Medicine II-Cardiology, Ulm University Medical Center.

### Statistical methods

Socio-demographic, lifestyle-characteristics, medical and laboratory results, and clinical characteristics (for ALS patients) were displayed in a descriptive way. Association of selected socio-demographic and clinical variables with serum levels of leptin, adiponectin and hs-CRP were compared using generalized linear models adjusted for case-control status, sex and age in order to appraise their distribution within the covariates. We modeled the geometric mean as the distributions were skewed to the right, and were approximately normal after log-transformation. The correlations with covariates were assessed with log-transformed biomarkers for the same reason.

Conditional logistic regression models were used to investigate the association between quartiles of leptin, adiponectin and hs-CRP and ALS. In analyses, data of 269 ALS cases and 492 control subjects with full set of covariates were included. Odds ratios (OR) and 95% confidence intervals (CI) were calculated stratified by sex and age groups. Quartile cut-points were calculated based on the sex-specific distribution in controls (Supplemental Table 1). The models were adjusted for relevant covariates such as smoking, educational level, occupational work intensity, family history of ALS and BMI.

In ALS-cases, time-to-death analyses were performed using Kaplan–Meier method and Cox proportional hazard models. Hazard ratios (HRs) were calculated to assess the prognostic value of leptin, adiponectin and hs-CRP serum concentrations for overall survival. Overall survival was defined as the time between baseline visit and death. As leptin, adiponectin and hs-CRP concentrations were skewed to the right we log-transformed these variables prior to analyses. For the graphical presentation the results were back transformed. Potential confounders included sex, age, diagnostic delay, site of onset and ALS-functional rating scale (FRS) at baseline. In addition, we modeled the median survival time using a Weibull survival model applying restricted cubic splines with knots at the 5, 50 and 90% percentiles. All provided p-values are two-sided. The statistical software package SAS release 9.4 (SAS Institute, Cary, NC, USA) was used.

## Results

The study sample comprised data of 289 ALS patients (59.5% men) with a mean age of 65.7 (SD 10.5) years and of 506 controls (59.5% men) with the mean age 66.3 (SD 9.8) years (Table 1). Compared to the control group, we observed in ALS patients a lower level of school education, lower smoking prevalence but higher levels of a positive family history of ALS. In relation to energy metabolism, it is noteworthy that ALS patients displayed lower BMI, and higher frequency of physical intense occupational working history. Compared to the controls median concentrations of leptin were lower in ALS patients (7.3 vs. 9.7 ng/L), while median concentrations of adiponectin and hs-CRP were higher (9.6 vs. 7.5 µg/L and 1.31 vs. 1.14 mg/L, respectively). Compared to ALS cases, higher concentrations of leptin and lower concentrations of adiponectin were found in both men and women among the control group, while for hs-CRP no difference was seen (Table 1). Most ALS-cases had a lumbar onset and the mean ALS-FRS was 39.0.

**Table 1:** Main characteristics of ALS patients and control subjects.

	N_Cases_	ALS-cases	N_Controls_	Control subjects
Age (years), mean (SD)	289	65.7 (10.5)	506	66.3 (9.8)
Sex	289		506	
Male, N (%)		172 (59.5)		301 (59.5)
School education, N (%)	289		503	226 (44.9)
<10^th^ grade		161 (55.7)		277 (55.1)
≥10^th^ grade		128 (44.3)		
Smoking	286		505	244 (48.3)
Ever, N (%)		132 (46.2)		
BMI (kg m^−2^), mean (SD)	289	24.6 (4.1)	404	26.5 (4.0)
Overweight (≥25 kg m^−2^), N (%)		115 (39.8)		303 (60.2)
Family history of ALS, N (%)	284		506	
Positive		11 (3.9)		2 (0.4)
Occupational work intensity, N (%)	278		500	
Light (mainly sitting)		100 (36.0)		233 (46.6)
Moderate (standing and walking)		115 (41.1)		203 (40.6)
Heavy (physically demanding)		63 (22.7)		64 (12.8)
Leptin (ng mL^−1^), median (Q1, Q3)	287	7.3 (3.7, 13.1)	504	9.7 (5.3, 19.9)
Adiponectin (µg mL^−1^), median (Q1, Q3)	289	9.6 (6.2, 15.0)	506	7.5 (5.2, 12.7)
hs-CRP (mg L^−1^), median (Q1, Q3)	289	1.29 (0.64, 3.22)	505	1.14 (0.65, 2.80)
*Clinical characteristics In ALS cases*				
Site of onset, N (%)	289			
Bulbar		91 (31.5)		
Cervical		73 (25.3)		
Thoracic		13 (4.5)		
Lumbar		97 (33.6)		
Uncertain		15 (5.2)		
Revised El Escorial criteria, N (%)	289			
Clinically suspected		48 (16.6)		
Clinically possible		36 (12.5)		
Clinically probable		92 (31.8)		
Clinically probable – lab. supported		83 (28.7)		
Clinically definite		30 (10.4)		
ALS-FRS, median (Q1, Q3)	288	39.0 (34.0, 42.5)		
Diagnostic delay (month), median (Q1, Q3)	288	5.0 (2.8, 9.0)		
Diagnosis to baseline visit (month), median (Q1, Q3)	289	3.6 (2.1, 5.9)		

The comparison of various sociodemographic data with clinical characteristics and the biomarkers revealed higher values of leptin and adiponectin in women and for adiponectin and hs-CRP in participants aged 65 years and above (Supplemental Table 2). Body mass Index (BMI) ≥25 kg/m^2^ was associated with higher leptin, higher hs-CRP and lower adiponectin values than BMI < 25 kg/m^2^.

**Figure 1:**
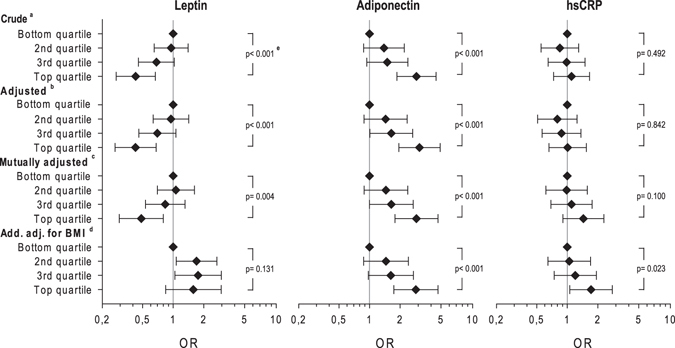
Adjusted Odds Ratios (ORs) for ALS with sex specific quartiles of leptin, adiponectin and CRP serum concentrations among 269 patients compared to respective controls. ^a^Stratified for age and sex. ^b^Additionally adjusted for school education, occupational work intensity, smoking (ever), family history of ALS. ^c^as ^b^but mutually adjusted for leptin, adiponectin and hs-CRP. ^d^as ^c^but additionally adjusted for Body mass index (BMI). ^e^P-values indicate trend over quartiles.

If analyses were controlled for age and sex (Fig. 1), inverse associations were observed between serum leptin concentrations and ALS. These associations were still present after further adjustment for family history of ALS, school education, occupational work intensity, smoking status, adiponectin and hs-CRP levels. Compared to the bottom quartile of leptin, the OR for risk of ALS was 0.49; (95% CI 0.29–0.80, p for trend 0.004) in the top quartile (Table 2 and Fig. 1). Additional adjustment for BMI for the association between leptin and ALS risk shifted the ORs over one, and test for trend was no longer statistically significant, indicating that leptin should be considered an intermediate factor on the causal pathway (and therefore BMI should not be included in the main model). The partial Spearman’s correlation coefficient between serum leptin concentration and BMI was 0.66 (p-value < 0.001).

**Table 2:** Odds ratios for ALS stratified for age, sex and adjusted for school education, occupational work intensity, smoking (ever), family history of ALS and mutually adjusted for serum concentrations of leptin, adiponectin and hs-CRP.

Odds ratio (95%-CI)	
Leptin* (N_Cases_ = 269, N_Controls_ = 492)	
Bottom quartile	(ref.) 1.00
2^nd^ quartile	1.07 (0.70, 1.62)
3^rd^ quartile	0.84 (0.53, 1.31)
Top quartile	0.49 (0.29, 0.80)
p-value for trend	0.004
Adiponectin (N_Cases_ = 269, N_Controls_ = 492)	
Bottom quartile	(ref.) 1.00
2^nd^ quartile	1.45 (0.89, 2.38)
3^rd^ quartile	1.64 (1.00, 2.69)
Top quartile	2.89 (1.78, 4.68)
p-value for trend	<0.001
hs-CRP (N_Cases_ = 269, N_Controls_ = 492)	
Bottom quartile	(ref.) 1.00
2^nd^ quartile	0.99 (0.62, 1.58)
3^rd^ quartile	1.10 (0.69, 1.76)
Top quartile	1.44 (0.91, 2.29)
p-value for trend	0.100

*Sex-specific cut-points.

Regarding serum adiponectin, in the mutually adjusted model increased levels were associated with ALS diagnosis (vs. bottom quartile OR 2.89; 95% CI 1.78–4.68, p for trend <0.001). Further adjustment for BMI did not substantially change the estimates. For hs-CRP the mutually adjusted model revealed no statistically significant association with ALS. However, analyses further adjusted for BMI showed increased risk of ALS for the top quartile of hs-CRP.

**Figure 2:**
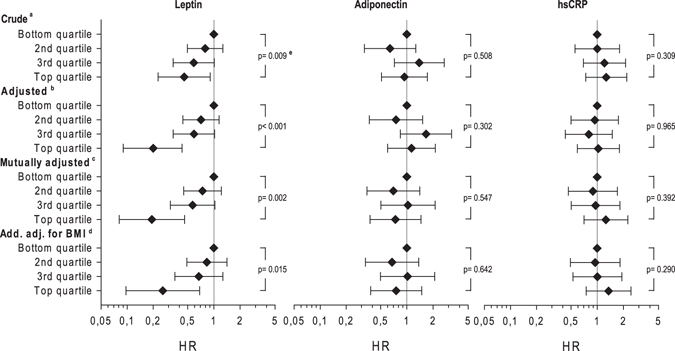
Hazard ratio for mortality by sex specific quartiles of leptin, adiponectin and hs-CRP serum concentrations among 279 ALS patients. ^a^Stratified for age and sex. ^b^additionally adjusted for age, diagnostic delay, site of onset and ALS-FRS. ^c^as ^b^but mutually adjusted for leptin, adiponectin and hsCRP. ^d^as ^c^but additionally adjusted for Body mass index (BMI). ^e^P-values indicate trend over quartiles.

As displayed in Table 3, 104 deaths among 279 ALS patients were identified during a median follow-up of 14.5 months. A shorter diagnostic delay and lower ALS-FRS status was evident in subjects who deceased (Table 3). The Kaplan-Meier curves by quartile showed better survival for patients with increased leptin levels, but the univariate analysis revealed no statistically significant differences in survival for all markers according to quartiles of exposure (Supplemental Figure 1). However, after adjustment for age and sex Cox proportional hazards models showed a clear inverse association between leptin levels and mortality (top versus bottom quartile HR 0.20; 95%CI 0.09–0.43; p for trend <0.001) (Fig. 2). Further adjustment for the other biomarkers and BMI revealed similar estimates. For adiponectin and hs-CRP no association with mortality was found in age and sex adjusted analyses (p for trend 0.302 and 0.965, respectively).

**Table 3:** Characteristics of ALS Patients (N = 279) with mortality follow-up by survival status.

	N_Deceased_	Deceased (N = 104)	N_Survived_	Survived (N = 175)
Age (years), mean (SD)	104	68.9 (10.3)	175	63.9 (10.2)
Sex				
Male, N (%)	104	56 (53.9)	175	114 (65.1)
BMI (kg m^−2^), mean (SD)	104	24.0 (4.2)	175	24.8 (4.0)
Leptin (ng mL^−1^), median (Q1, Q3)	104	7.2 (3.5, 13.3)	173	7.3 (3.8, 12.3)
Adiponectin (µg mL^−1^), median (Q1, Q3)	104	10.2 (7.5, 15.2)	175	9.2 (5.8, 14.5)
Hs-CRP (mg L^−1^), median (Q1, Q3)	104	1.64 (0.70, 3.18)	175	1.17 (0.61, 3.29)
Diagnostic delay (month), median (Q1, Q3)	104	3.0 (2.0, 6.0)	175	6.0 (3.0, 9.0)
ALS-FRS, median (Q1, Q3)	103	36 (32, 41)	175	41 (37, 43)

The prognostic value of leptin concentrations was additionally evaluated by using restricted cubic splines (Supplemental Figure 2) in order to appraise the influence on survival time by sex. Increasing leptin concentrations at baseline were positively associated with survival with a steeper slope in men than in women.

## Discussion

In this population-based case-control study, leptin serum concentrations were inversely associated with the risk of ALS as well as with survival during follow-up. Adiponectin serum concentrations were positively associated with ALS risk only. These associations showed a clear dose-response relationship and persisted after adjustment for potential confounders. For hs-CRP as a marker of systemic inflammation, neither an association with ALS risk nor with survival was evident, indicating that inflammation does not play a major role.

Our observations are in line with results from clinical observations and epidemiological studies showing that BMI is associated with ALS onset and progression^4^. O’Reilly *et al*.^17^ in a longitudinal study found that lower pre-diagnostic BMI was associated with increased risk of ALS^17^. Results from the EPIC cohort suggest that increased body weight was associated with decreased ALS mortality^18^. Increased lifetime physical activity was found to increase ALS risk in some studies^27^. Though the hypothesis that high energy expenditure increases ALS risk is biologically plausible, the overall evidence for an association of the risk of ALS with physical activity is weak and it cannot be excluded that other factors such smoking or alcohol consumption have biased previous associations^28^. In our study more ALS patients reported history of higher occupational work intensity than controls. However, adjustment for smoking status and occupational work intensity as indicator for physical activity did not substantially influence the estimates.

High levels of adiponectin were associated with higher risk of ALS. Adiponectin has strong anti-inflammatory properties and influences insulin sensitivity and fatty acid oxidation^29^. Our observation of no association between adiponectin and mortality is in line with findings from a clinical trial with pioglitazone, which modulates the transcription of insulin- sensitive genes and reduces insulin resistance in peripheral tissues. In this trial the application of pioglitazone deceased glycaemia and increased circulating adiponectin in ALS patients, but did neither influence BMI nor survival^30^. Thus, increasing adiponectin did not appear sufficient to improve survival in patients. Hs-CRP was neither associated with onset nor with prognosis of ALS. In a case-control study including 303 ALS patients recruited in a single medical center and 2,100 population-based controls no association was found between hs-CRP and ALS^12^. Our observation is consistent with a large-scale Mendelian randomization study including more than 4,000 ALS cases and 8,000 controls, showing no association between CRP-related single nucleotide polymorphisms (SNPs) and ALS onset^31^. Hs-CRP is an established biomarker for inflammation and we observed a moderate inverse correlation with ALS-FRS (Supplemental Table 3). However, other inflammatory biomarkers such as interleukin (IL)-6, IL-2, ferritin and tumor necrosis factor (TNF) maybe more important in this context^32^.

Our observations are consistent with the hypothesis that impaired energy metabolism is linked to the pathogenesis of ALS^4^. Leptin controls energy balance and acts as a pro-inflammatory adipokine modulating also the immune system^33^. Physiologically blood leptin regulates appetite and thermogenesis in the central nervous system^33^, in coordination with multiple other factors, in particular glucose and insulin. In our study, high leptin levels were associated with lower risk of ALS onset and longer survival of ALS patients. In the univariate Cox regression models, age (inversely), sex (male favorable) and ALSFRS-R (positively) were associated with survival (Supplemental Table 4). However, further adjustment for age, gender, site of onset and change of ALSFRS-R these factors did not substantially change the risk estimates for leptin. Interestingly, additional adjustment for BMI changes the direction of the leptin and ALS onset relationship, suggesting that the relationship of leptin is indeed caused by its strong correlation with fat mass, and thus indirectly reflects the protection offered by increased energy stores. This interpretation is consistent with leptin haploinsufficiency, that triggers increased body weight, being protective in mutant SOD1 mice^34^. This would be also coherent with other case-control studies showing an inverse association between polyunsaturated fatty acids and risk of ALS and increased energy intake in ALS patients^22^, and with the results from a small randomized clinical trial suggesting that diet high in lipids may have beneficial effect on the prognosis^10, 22^. However, further studies are necessary to disentangle the interrelation between these factors, and determine whether leptin could have an effect per se. Indeed, besides being correlated with fat mass, leptin increases fatty acid oxidation^33^. Thus, it cannot be excluded that high leptin levels result in increased lipolysis contributing to better prognosis of ALS patients with high leptin levels. Last, leptin has been shown to display neuroprotective potential, and this could contribute to the observed relationship between leptin and survival in our study^35^. To the best of our knowledge this is the first study investigating the association of adipokines with the onset and prognosis of ALS. However, measurements of additional metabolites and (neuro-) transmitters are necessary to further elucidate ALS pathogenesis.

Strengths of our study are its large sample size with virtually complete follow-up of the ALS patients. It should be critically evaluated that the response among our control population was relatively low, however, we selected a clear population-based approach instead of selecting controls in a hospital setting and in the final analysis we carefully matched for gender, age as well as geographic region and used multivariable analysis to further adjust for potential confounders. Finally, the case-control design of the analysis on ALS onset limits the causal interpretation of the results as the biomarkers were measured after clinical diagnosis of ALS and not at or respectively before disease initiation. Nevertheless, this argument is less important for assessing the prognostic value within the prospective cohort approach of our study. Moreover all biomarkers were measured in one laboratory according to a standard protocol under blinded conditions. It could be considered as a limitation that no dietary data was available for the current analyses. However, energy dense diets are likely taken into account through adjustment with BMI. Though the recruitment of study participants in Southern Germany is well described and the epidemiological characteristics of the study population are in the range of other studies, this should be considered when generalizing the results. Although our control group had higher BMI and duration of school education, these factors were adjusted in the final analyses. In addition, since higher social status is associated with lower BMI, the associations in the case-control study could be even underestimated. When interpreting the results it should be considered that biomarker measurements were only done at a single time point and part of the association in the case-control part may represent reverse causality. Therefore, corroboration is urgently needed within a prospective study.

In summary, our case-control study showed that serum concentrations of leptin and adiponectin are associated with the risk of ALS. The additional survival analysis in ALS-cases revealed only for leptin a clear positive association with prognosis. These results suggest that ALS onset as well as prognosis appear to be related to energy homeostasis.

## Electronic supplementary material

Supplementary Information
