# Supplementary material for: Adipokines, C-reactive protein and Amyotrophic Lateral Sclerosis – results from a population- based ALS registry in Germany
Source: Sci Rep. 2017 Jun 29;7:4374. doi: 10.1038/s41598-017-04706-5 (PMC5491500; doi:10.1038/s41598-017-04706-5)
Supplement: Supplementary file 1 — Supplementary Information [file 41598_2017_4706_MOESM1_ESM.pdf]

Supplement to

Adipokines, C-reactive protein and Amyotrophic Lateral Sclerosis – results from a population-based ALS registry in Germany

Gabriele Nagel[1], Raphael S Peter[1], Angela Rosenbohm[2], Wolfgang Koenig[3,4,5], Luc Dupuis[6], Dietrich Rothenbacher[1], Albert C Ludolph[2]

[1] Institute of Epidemiology and Medical Biometry, Ulm University, Ulm, Germany
[2] Department of Neurology, Ulm University, Ulm, Germany
[3] Department of Internal Medicine II - Cardiology, University of Ulm Medical Centre, Ulm, Germany
[4] Deutsches Herzzentrum München, Technische Universität München, Munich, Germany
[5] DZHK (German Centre for Cardiovascular Research), partner site Munich Heart Alliance, Munich, Germany
[6] INSERM U1118, Université de Strasbourg, Strasbourg, France

Supplemental Table 1. Sex specific biomarker quartiles

	Leptin (ng mL^{-1})	Adiponectin (µg mL^{-1})	hs-CRP (mg L^{-1})
		**Men**	
Bottom quartile	-3.58	-4.50	-0.55
2nd quartile	>3.58-6.91	>4.50-5.99	>0.55-1.13
3rd quartile	>6.91-11.69	>5.99-8.84	>1.13-2.89
Top quartile	>11.69-	>8.84-	>2.89-
		**Women**	
Bottom quartile	-9.01	-7.12	-0.64
2nd quartile	>9.01-18.59	>7.12-11.99	>0.64-1.17
3rd quartile	>18.59-30.89	>11.99-15.89	>1.17-2.65
Top quartile	>30.89-	>15.89-	>2.65-

Supplemental Table 2. Association of selected sociodemographic and clinical variables with leptin, adiponectin and hs-CRP serum values

		Leptin (ng mL^{-1})		Adiponectin (µg mL^{-1})		hs-CRP (mg L^{-1})	
	N	geometric mean	p-value	geometric mean	p-value	geometric mean	p-value
Age (years)							
< 65	336	7.8		7.2		1.17	
≥ 65	455	9.0	[a]0.11	9.2	[a]<0.001	1.49	[a]0.004
Sex							
Male	469	5.8		6.6		1.38	
Female	322	14.8	[b]<0.001	11.5	[b]<0.001	1.29	[b]0.36
BMI (kg m^{-2})							
< 25	373	5.4		9.8		1.00	
≥ 25	418	12.7	[c]<0.001	7.2	[c]<0.001	1.76	[c]<0.001
School education							
< 10th grade	385	8.3		8.3		1.61	
≥ 10th grade	403	8.6	[c]0.92	8.3	[c]0.21	1.13	[c]<0.001
Smoking							
Never	415	8.8		8.7		1.33	
Ever	372	8.1	[c]0.25	7.8	[c]0.75	1.33	[c]0.98
Occupational work intensity							
Light	331	8.6		8.5		1.25	
Moderate to heavy	443	8.4	[c]0.40	8.1	[c]0.19	1.43	[c]0.20

[a] adjusted for case-control status, and sex
[b] adjusted for case-control status, and age
[c] adjusted for case-control status, sex and age

Supplemental Table 3. Pearson correlations of age at baseline, ALS-FRS and diagnostic delay with biomarkers

		Leptin [a]	Adiponectin [a]	hs-CRP [a]
Age	r	0.027	0.281	0.150
	p	0.65	<0.01	0.01
ALS FRS	r	-0.126	-0.117	-0.227
	p	0.03	0.05	<0.01
Diagnostic delay	r	0.040	0.068	-0.012
	p	0.50	0.25	0.84

[a] Log transformed

Supplemental Table 4. Univariate associations with survival in ALS cases

	HR (95% confidence interval)
Sex	
Male	0.72 (0.49, 1.06)
Age (years)	1.04 (1.02, 1.06)
ALS FRS	0.94 (0.92, 0.96)
Onset site	
Bulbar	0.99 (0.66, 1.48)
Diagnostic delay (month)	1.00 (0.96, 1.05)

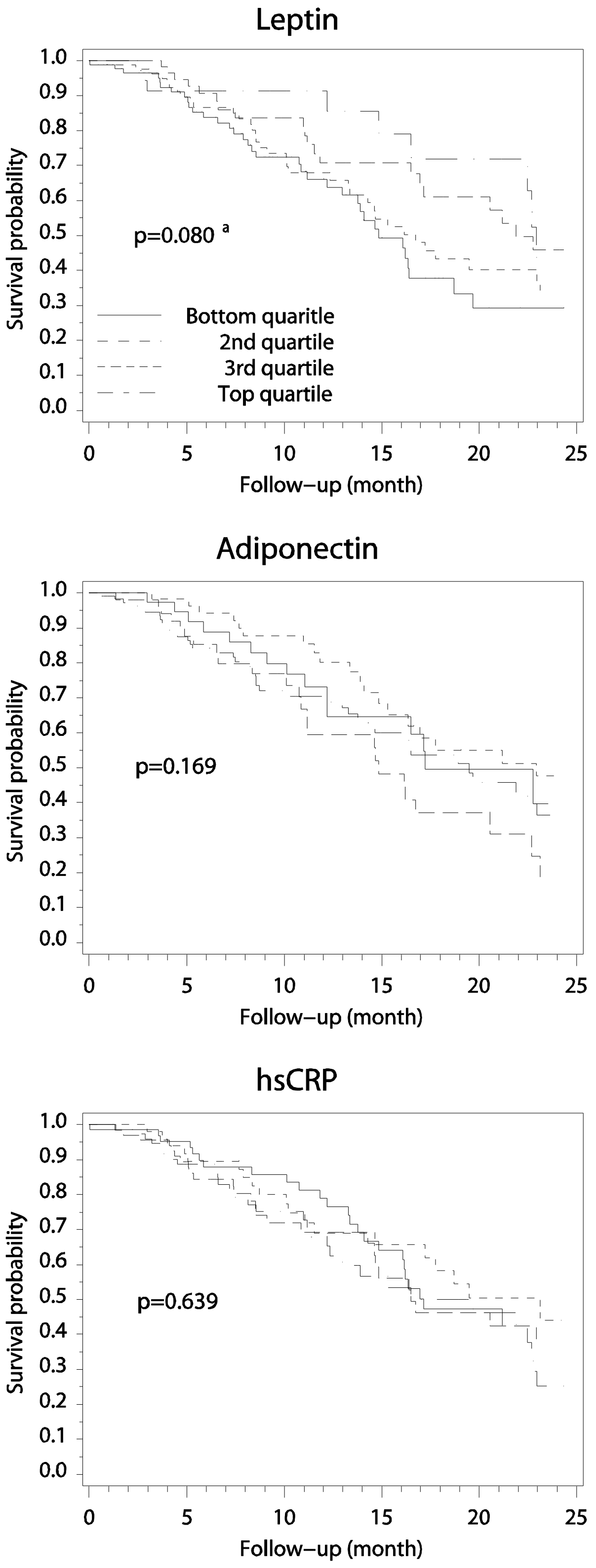

Supplemental Figure 1. Unadjusted survival probability by sex specific quartiles of leptin (N=277), adiponectin (N=279) and hs-CRP (N=279) ALS patients

[a] log-rank test p-value for equality over quartiles

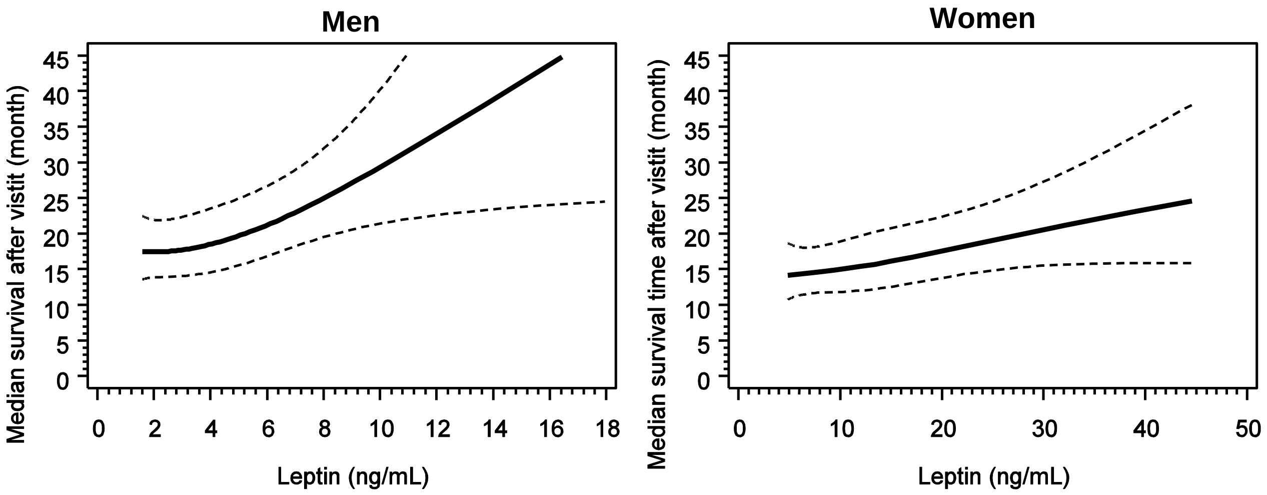

Supplemental Figure 2. Median survival time (bold line) (with 95% confidence band (dotted lines)) of ALS patents after baseline visit in month by leptin levels, adjusted for age, diagnostic delay, site of onset and ALS-FRS.
